# The concept of the schizophrenic lifeworld revisited

**DOI:** 10.3389/fpsyt.2025.1625364

**Published:** 2025-09-26

**Authors:** Marco Kramer, Paraskevi Mavrogiorgou, Georg Juckel

**Affiliations:** LWL University Hospital Bochum, Department of Psychiatry, Psychotherapy and Preventive Medicine, Ruhr University Bochum, Bochum, Germany

**Keywords:** schizophrenia, lifeworld, phenomenology, psychopathology, Husserl, Blankenburg, phenomenological psychopathology

## Abstract

Disturbances regarding the lifeworlds of patients with mental disorders are recurrently mentioned in psychiatric literature, but the concept often remains poorly defined and unclear. The present article takes the lifeworld in schizophrenia as an example to elucidate this concept. First, the concept “lifeworld” is traced back to its philosophical roots. The classical phenomenology particularly of Edmund Husserl is revisited to establish its general meaning. Secondly, synthesizing classical and current phenomenological-psychopathological literature with empirical findings, we explore different interpretations regarding schizophrenia and their relation to existing theories: (1) a general loss of lifeworld through diminished natural self-evidence, (2) the constitution of an idiosyncratic private world (*Eigenwelt*), (3) the perception of a strange and alien world (*Fremdwelt*), and (4) schizophrenia as part of our shared intersubjective lifeworld, albeit with a particular vulnerability to social passivity and exclusion. We argue for a pluralistic understanding that situates schizophrenic lifeworlds on a spectrum between isolation and participation, between radically anomalous experiences and shared lifeworlds. Therapeutically, the analysis highlights the importance of establishing bridges of shared self-evidence, attentive presence, and supportive milieus, while acknowledging the protective and emancipatory functions of private worlds. More broadly, acknowledging schizophrenic lifeworlds as embedded in, yet challenging, our shared world opens new directions for research and calls for a more inclusive psychiatry.

## 1 Introduction

“The patient is ill; this means that his world is ill” – J.H. van den Berg (1)

Social neuroscience and psychiatry increasingly focus on the social-cultural and environmental determinants of mental health. It becomes increasingly clear that the etiology and course of mental disorders is not merely dependent on neurobiological factors, but at least as much on the interactions of a person within their lifeworld (2, 3). Trending research topics include the impact of lived environments such as urban contexts (4), the digitalization of our lifeworld (5), or the self-world relationship in specific mental disorders such as schizophrenia or autism spectrum disorder (6). The term “lifeworld” is repeatedly used in these contexts, yet outside phenomenological psychiatry it often remains poorly defined.

Thus, this article seeks to clarify the psychiatrically relevant meaning of “lifeworld”. On the one hand, it sheds light on its theoretical underpinnings in phenomenology. This philosophical discipline was founded by Edmund Husserl (1859-1938) and deals with an unprejudiced analysis of the core structures of our lived experience. It especially focuses on pre-reflective processes regarding self, spatiality, corporeality, temporality and interpersonality. On the other hand, this article illustrates the relevance of the “lifeworld” for psychiatry by synthesizing empirical studies and phenomenological psychopathology. Our focus will be on schizophrenia, which has long been central to phenomenological psychopathology due to its profound alterations of lived experience. It is also particularly relevant due to its heavy burden on society and its healthcare systems (7).

Inspired by phenomenology, phenomenological psychopathology aims at peeling out the core of schizophrenia, the “trouble générateur” (8) or “basal disturbance” (*Grundstörung*) (9). It is assumed that this psychopathological core underlies its diverse clinical manifestations, including delusions, hallucinations, disorganized speech and negative symptoms (e.g. lack of drive, disorganized behavior). In recent decades, disturbances of mineness or ipseity, also called “minimal self disorder”, have become the dominant hypothesis (10–12), closely linked to a “temporal fragmentation of self-experience” (13). Disturbances of the intersubjective lifeworld, by contrast, were deemed secondary to these intraindividual factors.

In line with previous calls for a pluralistic approach to basal disturbances (14, 15), this paper discusses four major interpretations of the schizophrenic lifeworld:

1. as a “loss of self-evidence” (9) and thus of lifeworld, in line with the works of Blankenburg who has conceptualized schizophrenia mainly as a disorder of intersubjectivity (16),
2. as a “lifeworld of solipsism” (17), i.e. a private or solipsistic world,
3. as a world estranged “from our everyday experience and the world” (18), which is closely linked to accounts of a minimal self disorder, and
4. as a still-participating but marginalized part of our shared intersubjective world.

We argue that, despite individual deficits, the intersubjective dimension of schizophrenia must not be neglected. A purely individualizing and deficitary perspective, which decontextualizes affected persons from their practical-social life processes (16), risks reinforcing their vulnerability to social passivity and exclusion. The following sections will first review the concept of the lifeworld in phenomenology, then analyze its interpretations in schizophrenia, and finally discuss the implications for clinical practice, research and society.

## 2 Lifeworlds and anomaly

### 2.1 The Husserlian concept of lifeworld

Any attempt to clarify the term “lifeworld” for the psychiatric context quickly encounters obstacles. Lifeworld is a complex, inconsistently defined concept. Husserl develops it throughout his research manuscripts and, more systematically, in his *Crisis* (19). However, a comprehensive ontological definition of all the contents comprised by our lifeworld, or the role of anomalies within a lifeworld have never been accomplished and are not within the scope of this article. Still, Husserl and his interpreters have provided us with enough material to capture the most important characteristics of the lifeworld.

Generally speaking, the lifeworld is the world “in which we live” (20). This seems tautological and not very informative at first glance. It was specified by Husserl in several ways. As a world of experience, the lifeworld is “constantly valid for us, with unquestioned certainty, [ … ] simply there” (19). It is the world that surrounds us, which “is always already pregiven to us” and provides us with “everything which becomes the substrate of a possible judgment” (20). As such, the lifeworld is the intentional background of all our experiences. Being the “universal field of establishable facts” (19), it provides the horizon of what can ever be experienced according to our individual and human disposition. Beyond experience, however, the lifeworld is also the universal field of all further intentional actions. It is the world “in which we live, move, communicate and theorize” (21). It is the “ground of all our interests and life-projects” (19), the world in which “we carry out activities of cognition and judgment” (20), the world of our everyday life processes.

In short, Husserl’s concept of the lifeworld designates it as horizon of experience, the background world that is always already given, taken for granted, and in which all our practical, cognitive, and social activities are grounded.

### 2.2 Self-evidence and typification

Further differentiation makes this term more tangible. Lifeworld encompasses all that which remains self-evident and unthematized in our everyday lives. It is the source of all our habitual and taken-for-granted assumptions (20). It is the “ultimate source of primal self-evidence” and our “natural attitude” (19), i.e. our ordinary way of life in which we live automatically, and take things for granted without deeper reflection or justification. In this sense, “self-evidence” (*Selbstverständlichkeit*) refers not to a rational proof but to the tacit, pre-reflective certainty with which the world appears as familiar and trustworthy. It denotes the background confidence that things are “simply there” and make sense without requiring explicit justification. For example, we normally do not question that a chair will support us when we sit down. This background of unquestioned obviousness makes ordinary action possible until it is disrupted, for instance, in the anomalous experiences of schizophrenia.

On the one hand, this self-evidence includes our brute spatiotemporal environment with its pregiven spatial configuration and temporal flow. We inhabit it naturally and navigate it as embodied agents in our everyday lives. On the other hand, lifeworld encompasses our habitual typifications. This means that we superimpose our environment with types (e.g. a furry four-legged friend with the type dog) and corresponding experiences, expectations and values (e.g. that we are not surprised if it barks but would be quite irritated if it meowed). Our lifeworld encompasses all these taken-for-granted assumptions. They allow us to form heuristic predictions and to develop a fundamental trust in and “familiarity-with” the world (22, 23).

In short, the lifeworld sustains a basic sense of self-evidence: the tacit confidence that the world is familiar and predictable. Through habitual typifications, this certainty provides orientation for everyday action, until it is disturbed, as in anomalous psychotic experiences.

### 2.3 Intersubjectivity, sedimentation, and tradition

This typification is ultimately a result of intersubjectivity (19). It is important to note that Husserl does not conceptualize the lifeworld as confined to isolated subjects or our individual lived experiences. Our lifeworld’s taken-for-granted assumptions are not only formed through gradual exploration of our environment from infancy, but also always constituted in a community and its “communalization” (19) of experiences. What we take for granted depends on what we agree on with others, even if never explicitly negotiated.

From infancy onwards, our lifeworld is constituted in a reciprocal interplay between the individual and their environment, which they share with primary caregivers such as parents, role models and teachers (23). Interaction with others enables the validation of knowledge and the commitment to shared norms and values. Thus, our lifeworld is always already rooted in a sediment of other people’s experiences. This sediment comprises the tradition of norms and practices within a specific historical community. Through habitualization and socialization these become part of our individual store of knowledge (24). Our lifeworld is thus significantly shaped by socio-cultural and historical factors and never born “*ex nihilo* from ourselves” (15). Thus, “sedimentation” means that past experiences and practices are culturally retained and continue to shape present perception and understanding, often without explicit awareness.

Hence, lifeworld is never purely individual but always socially and historically mediated: it arises through interaction, communal validation, and the sedimentation of traditions that continue to shape present experience.

### 2.4 Normality and anomality

The term “normality” is closely related to the normal typification and taken-for-granted assumptions within our shared lifeworld. Husserl characterized normality with the concepts of concordance, optimality, and averageness (25–27): Concordance refers to a coherence between present and prior experiences on an individual level, but also to a coherence between individual and intersubjective experiences. Accordingly, normal is that which is coherent to our past experiences and what we can agree on with other individuals. Optimality refers to the suitability of an experience to achieve the aims of a perception or action, both on an individual and on an intersubjective level. Averageness refers to that which does not deviate too far from a typical range of experience and behavior (25).

Strikingly, according to Husserl, the concept of normality in the sense of averageness already includes the concept of anomaly. Anomaly is understood as deviation from this normality. According to Husserl, it can take the forms of “over-normality” due to special skills or “under-normality” due to disease (25). Thus, in general, Husserl deems anomaly, like normality, “an essential structural component of every world” (25). Normality and anomaly are mutually constitutive: unquestioned normality is only established through the “intrusion of the anomalous” (28) while individual deviations are always anomalous in relation to a normative or statistical norm (29). Both are dynamic processes that depend on standardization processes (30). Husserl’s characterization of the lifeworld as a “universe of given self-evidences” (19) must therefore be supplemented by all kinds of what is not self-evident.

This dialectic relationship between self-evidence and non-self-evidence is constitutive for human being-in-the-world and was later taken up by Blankenburg in his analysis of the basal disturbance in schizophrenia (9). Thus, the horizon of the lifeworld necessarily extends into marginal areas of anomaly, which lie in a “fog of dark indeterminacy” (31), but which nevertheless remain principally accessible. Normality can thus be understood as a perceptual filter that anticipates experiences coherent with our own past, with others’ expectations, within a typical range, and aligned with our goals. At the same time, it retains an open horizon for anomalous experiences, that deviate from these experiences. This allow the intersubjective lifeworld to remain dynamically open to novelty.

In short, the lifeworld is both socially inherited and dynamically open: it rests on sedimented traditions and shared norms, yet remains flexible through the constant interplay of normality and anomaly. This tension between what is taken for granted and what appears as anomalous is crucial for understanding how experience can both stabilize and destabilize.

### 2.5 Plurality of lifeworlds

However, there remains a tension in Husserl’s work. In addition to this idea of an overarching intersubjective lifeworld, we can also conceive individual lifeworlds as our respective first-person “horizon within which we develop our practical knowledge and make sense of social norms, customs and expectations” (19). On the one hand, Husserl endeavors to extract the core of a universal lifeworld from all first-person experiences, so that the “talk of ‘life-worlds’ [in plural, A/N] would be a misunderstanding” (19). On the other hand, he recognizes that the lifeworld is “always and necessarily respectively mine” (25), i.e. always the concrete-historical lifeworld of a subject or a community of subjects with its concrete content of self-evident facts and traditions. Not only different communities, but also “meaning spheres” within these communities such as professional life or family life constitute their own lifeworlds, each with their own taken-for-granted assumptions (32).

This suggests the existence of a potentially infinite number of lifeworlds, each with its own norms and customs (33, 34), or as sociologist Alfred Schütz put it, “an infinite number of various orders of realities, each with its own special and separate style of existence” (35). The talk of a “schizophrenic lifeworld” only makes sense against such a framework of a plurality of lifeworlds.

### 2.6 Towards a concept of the schizophrenic lifeworld

Now, equipped with the basic conceptual framework, we can move on to the concept of the schizophrenic lifeworld. However, before we come to specific interpretations, a few qualifying remarks are necessary to prevent misunderstandings:

1. From Husserl’s concept the hypothesis could be derived that patients with schizophrenia produce their own intersubjective tradition with its own culturally sedimented normality. This assumption requires qualification. Indeed, Husserl discusses the thought experiment of “a people of the color-blind” (25) who constitute their own lifeworld and normality that deviates from the prevailing experience, as it does not contain specific colors and related idioms. In contrast, episodes of acute psychosis with idiosyncratic delusions rarely seem compatible with a coherent shared lifeworld, as they tend to be heterogeneous, self-referential, and difficult to validate intersubjectively (36). Outside of folie à deux, no two delusional beliefs are completely alike. Although this makes a “people of schizophrenics” as a cultural community with its own tradition difficult to conceive, it does not entail that people with schizophrenia cannot develop shared worlds. As recent user movements such as *Mad Pride* or *Recovery in the Bin* demonstrate, (ex-)patients, or so-called “survivors”, do in fact constitute communities, shared narratives, and cultural counter-worlds. These examples show that schizophrenic experiences, while sometimes radically individual, can also be integrated into collective traditions and forms of normality. Strikingly, this coexistence or rather, the dialectical intertwining, between idiosyncratic modes of experience on the one hand and, on the other, a community with its own taken-for-granted lifeworld assumptions not only creates tension, but also reflects a tension within Husserl’s own work. As Arthur Tatossian emphasized, classical Husserlian phenomenology suffered from an intermittence of phenomenological vigilance (37). In this regard, it is important not to marginalize psychotic experiences from a normocentric, deficit-oriented perspective, but vigilantly attend to them as phenomena in their own right. Applying the standpoint of Husserl’s generative phenomenology may then make it possible to analyze how such experiences sediment into collective narratives and shared traditions, as recent developments in user movements demonstrate. Taking this into account, we have to consider “schizophrenic lifeworlds” along a spectrum: from plural individual lifeworlds with overlapping but also idiosyncratic features, through partially shared lifeworlds e.g. within user movements, up to their embeddedness in the everyday lifeworld, from which they nevertheless deviate with regard to concordance, optimality and/or averageness.
2. It is a difficult challenge to define the boundary between schizophrenic lifeworlds and shared lifeworlds. The characterization of the schizophrenic and the shared lifeworld as “two partially overlapping circles” (38) certainly falls short to grasp the complexity of their relation. Against the backdrop of their dialectic intertwinement, it is impossible to define fixed boundaries between normality and anomality, between our shared lifeworld and the lifeworld in schizophrenia (33). Rather, the diversity of lifeworlds gives rise to endless possibilities of relating two lifeworlds, except that they cannot completely diverge but always share a core of experiences even if it’s only some brute spatiotemporal facts (33). Thus, our shared lifeworld can best be described as a complex network and chain of multiple lifeworlds, fields of meanings and normalities, that overlap and dissolve into one another (39).
3. In its focus on the subjective first-person experience and the transcendental ego, Husserl’s phenomenology certainly has problems satisfactorily explaining the understanding of others. This is even more true in cases of schizophrenia where some anomalous experiences are inaccessible even to those affected themselves, and more so to the phenomenologist or psychiatrist. Any phenomenological analysis thus remains preliminary and must carefully bracket pre-existent theoretical assumptions. Otherwise, it runs the risk to assume a questionable “monopoly of reason” (40), to quote Maurice Merleau-Ponty’s appropriate words here, and to pathologize divergent characteristics from an unjustified normocentric stance.
4. The symptoms of schizophrenia not only have a strong inter-individual but also intra-individual variability. They differ greatly between acute paranoid-hallucinatory syndromes and chronic syndromes dominated by negative symptoms. This makes general conclusions more difficult and highlights the importance of indicating which phase of the disorder the analysis refers to. For example, whereas Blankenburg explicitly focus on pauci-symptomatic forms of schizophrenia, Tatossian mainly focused on “full-blown” schizophrenia and both came to very different results regarding the basal disturbance underlying the disorder (15). In general, it remains dubious whether it is possible to “distinguish between the core of an illness and its immediate sequelae (which may be compensatory or consequential)” (41). Thus, the following interpretations of the “schizophrenic lifeworld” should not be understood as mutually exclusive, but rather as pluralistic approaches that are each justified for a certain scope. We will therefore treat them as complementary rather than competing models.
5. It should be borne in mind that the concepts of anomality and illness are not congruent (30). Instead, (mental) illness can be thought of as a special form of anomality, which is characterized, for example, as a lack of adequate responsiveness of an organism in its environment (42), which supports the intersubjective dimension of illness.

These clarifications suggest that the notion of a “schizophrenic lifeworld” should not be reduced to a single model or rigid boundary. Rather, it must be approached with modesty and attention to individual variability. On this basis, we can now turn to the different phenomenological interpretations of the schizophrenic lifeworld.

## 3 Loss of lifeworld in schizophrenia

### 3.1 Loss of self-evidence as basal disturbance

As a first interpretation, it is conceivable that a schizophrenic lifeworld primarily suffers from a loss of everyday self-evidence - up to a complete “withdrawal from the intersubjectively constituted lifeworld that we share” (9). This view is supported by severe alterations in experience such as delusional systems, disorganized speech, or perceptual anomalies, that reduce the overlap with our shared lifeworld. But on top of that, the normally seamless experience “of a continuously unfolding world” (43), which is the spatiotemporal core of any constituted lifeworld, may be fundamentally compromised.

Consequently, patients with schizophrenia might not be able to constitute a lifeworld at all. In line with this, Blankenburg describes a “loss of natural self-evidence” (9). While in a healthy psyche non-self-evident facts give rise to new forms of self-evidence in a dialectic process, Blankenburg characterizes the basal disturbance (*Grundstörung*) in schizophrenia by a disproportionate loss of natural self-evidence (*natürlichen Selbstverständlichkeit*) relative to what is not self-evident. As a result, meanings and references within the shared lifeworld lose their binding character for individuals with schizophrenia. This manifests in a disturbance of affectivity, described as a “void, indifference, and desolation of nearly all mental life” (9), which becomes visible as affective flattening, i.e. a near-absence of emotional resonance and disengagement from the intersubjective lifeworld.

Blankenburg argues that this affective flattening, especially if combined with a disruption of temporal orientation toward past and future, culminates in schizophrenic autism, i.e. the failure to constitute and participate in an intersubjective lifeworld. That way, affected persons lose their “intersubjective anchoring (*Verankerung*) into the lifeworld” (15). Another important nuance is Blankenburg’s distinction between the loss of “the self-evidence of the self-evident, not of the self-evident itself” (9). The basal disturbance of schizophrenia is not the loss of specific contents typically taken for granted, but a disconnection from any form of self-evidence (44), prior to conscious reflection. Thus, patients with schizophrenia may not only fail to take part in our shared lifeworld due to loss of its concrete self-evident norms but might also fail to constitute even individual regularities and norms. This can cause a general loss of “familiarity-with” the everyday world (22).

### 3.2 The loss of lifeworld

In the absence of such certainties, Blankenburg (1971/2012) alludes vaguely to a “private world, however poorly filled it may be with specific contents” (9). Yet this notion appears problematic. If self-evidence is lost to the point where only incoherent and fragmented beliefs remain, the result is a collapse of individual normality. Husserl illustrates such a radical “denormalization” (43) in a thought experiment describing the complete breakdown of the practical human environment (25). Even if a shared spatiotemporal core of experience remains, a shared “objective grasp of things” (9), Husserl argues that this cannot alleviate the existential distress of those who feel cut off from the intersubjective lifeworld (25). This results in desperate but ultimately failing attempts to adapt to the taken-for-granted structures of the lifeworld (9, 25).

This hypothesis thus implies a radical loss of lifeworld. However, this would render the concept of a “schizophrenic lifeworld” meaningless, since in such cases no lifeworld is constituted at all. In contrast to Blankenburg’s explicit focus on paucisymptomatic forms and early stages of schizophrenia (15), this radical loss seems more plausible in acute phases marked by severe incoherence in experience and behavior.

Still, two objections demand caution. First, Husserl’s notion of the universal character of the lifeworld implies that it is “not occasionally, but always and necessarily [ … ] given” (19), meaning that even individuals with severe anomalies constitute some kind of lifeworld. Second, assuming a loss of lifeworld risks precluding therapeutic engagement, since it denies any possible common ground of normality or self-evidence.

Accordingly, the “loss hypothesis” highlights how schizophrenia may threaten the very foundation of self-evidence and intersubjective anchoring. Yet it remains problematic: Husserl’s notion of the lifeworld as universally given, as well as the clinical experience that dialogue and a shared basis with patients are possible, are reason enough to take into account alternative interpretations, such as the notion of a private world.

## 4 A private world in schizophrenia

### 4.1 A world of idiosyncratic self-evidences

The concept of a schizophrenic private world (*Eigenwelt*) refers to a lifeworld that, beyond a spatiotemporal core, also possesses its own self-evidences and own peculiar normalities. From an outside perspective of the shared lifeworld, this private world may appear as a primarily inaccessible or ultimately incomprehensible strange world (*Fremdwelt*). It could constitute an entirely different, individual lifeworld - one that may overwhelm, delight, or confuse the affected subject.

This interpretation stands in contrast to the “loss of lifeworld” hypothesis discussed in Chapter 3. Rather than assuming a radical void, it emphasizes that individuals with schizophrenia still pursue complex purposes and develop taken-for-granted assumptions. In the case of delusional convictions these self-evidences might even reach a degree of *a priori* certainty. In this regard it is important to note that even in mental health, the degree of self-evidence anchored in our shared lifeworld varies greatly from individual to individual (9), so that deficits are not categorically pathological.

The compensatory struggle for self-evidence can become pathological, as in “morbid rationalism”: the often doomed attempt to explain unfamiliar practices by schematic, purely logical rules (45). Yet out of this process, new idiosyncratic self-evidences may emerge, sometimes in open rejection of socially shared norms, causing retreat into an eccentric stand (46). Even delusional and hallucinatory experiences can form part of new self-evidences, expanding the individual’s experiential horizon beyond what would have seemed possible before the disorder. Still, these new self-evidences are usually intermingled with the sediment of the intersubjective lifeworld in which the affected individuals were originally socialized. Delusional contents often depend on cultural sediments, such as scientific or technological progress (47). Furthermore, patients frequently act in ways that remain anchored in residual shared self-evidences, even while holding contradictory beliefs, an ambivalence described as “double bookkeeping” (48).

Thus, the notion of a schizophrenic private world highlights that individuals do not simply lose lifeworld, but rather reconstitute it through new, idiosyncratic self-evidences. These may be eccentric or pathological, yet they remain intertwined with cultural sediments and residual structures of the shared lifeworld.

### 4.2 The limits of solipsism

Building on the minimal self-disorder hypothesis, a diminished attunement to the shared world’s self-evident meanings has been proposed (11). When this gap to shared normality can no longer be reflexively bridged, individuals may become confined to their own subjective sensory and affective experiences (18). They appear as solipsists, trapped within a self-contained private world revolving exclusively around one’s inner experiences (17). This notion of a private world (*Eigenwelt*) closely resonates with Bleuler’s concept of schizophrenic autism: a detachment in which “the inner life assumes pathological predominance” and patients “live in a world of their own” where they “have encased themselves with their desires and wishes” and “cut themselves off as much as possible from any contact with the external world” (49). The various disturbances in fundamental mental capacities foster a retreat into the nucleus of an internal stage. It overlaps with the shared lifeworld only at the level of a minimal spatiotemporal experiential core, yet one that bears radically different structures of implicit meaning. The notion of a private world (*Eigenwelt)* thus points to the paradox of a world no longer shared yet still experienced as if intersubjectively valid.

However, it would be misleading to absolutize this concept. Lifeworld, by its very nature, possesses a necessary intersubjective dimension and cannot be conceived as a purely idiosyncratic, self-contained world. Moreover, the minimal self must itself be understood as inherently intersubjective and social (50). This is supported by pre-linguistic and pre-reflective forms of social interaction, beginning even before birth through responses in the womb, reactions to voices, and, later, in infancy through implicit proto-conversations and gaze following, long before linguistic interaction develops (50). Thus, our most basic sense of self, the minimal self, is relational from the outset. It is never purely solipsistic, but always already constituted in relation to others. Husserl’s thought experiment of a “solipsistic subject” underscores this point: such a subject would be “anomalous, insane, to the point that it no longer constitutes a world” (33). In fact, solipsism like death would lead to a “dissolution of the appearance-type of material objectivity [ … ] which alone makes possible the co-presence of the psychical within a regulated psychophysical correlation,” resulting in an “exit from the world” (33).

Thus, a “solipsistic private world” would no longer be a genuine lifeworld but radical solipsism marks the collapse of world-constitution itself. The term solipsism may thus function as a metaphor for the loss of world. It might further be useful to express the paradox that individuals affected by schizophrenia absolutize their inner experiences without being able to reflect on their increasing disconnection from the intersubjectively shared taken-for-granted assumptions (17). However, they are disconnected in a practical and not in an ontological sense.

### 4.3 Private worlds within the shared world

Instead, the notion of a private world also resonates with Heidegger’s account of affective attunement (*Befindlichkeit)* (51) and its Daseinsanalytic elaboration by Ludwig Binswanger (52). In contrast to the social world (*Mitwelt*) and the surrounding physical world (*Umwelt*), *Eigenwelt* refers to the immediate lived experience through which individuals encounter themselves in situations (53). Yet, as Heidegger stressed: “*Dasein* [literally ‘being-there’ …] does not somehow first get out of an inner sphere in which it has been proximally encapsulated, but its primary kind of Being is such that it is always ‘outside’ alongside entities which it encounters and which belong to a world already discovered.” (51). This means that our world-relatedness can in principle not be reduced to a private world, but this private world is always already tightly interwoven with an intersubjectively accessible world. A private world (*Eigenwelt*) is therefore best understood as a “center surrounded by further horizons” (54): the private sphere of felt experience and thought, which is intertwined with a sediment of norms and taken-for-granted assumptions drawn from the shared lifeworld.

In that sense, each individual that constitutes a lifeworld also constitutes their own private world (*Eigenwelt*), which is not that private at all, but is part of a shared world of meaning. Thus, even anomalous lifeworlds remain inescapably embedded within an intersubjective context and its shared structures of normality.

In this regard, a recent qualitative study found that many patients with schizophrenia describe a diffuse sense of “double reality” that seems to precede the phenomenon of double bookkeeping (55). Participants frequently reported feeling simultaneously connected to the shared world and to a separate, often more profound reality containing hallucinatory and delusional experiences. For example, some stated: “I live in the world everybody else does, where we know that the table is a table, and then in my own world, where I have visions and hear voices”; others noted that “there is a common reality that we share, and then I can tap into this other reality,” or described themselves as “living between two worlds [ … ], my own little world and then the surrounding world. And I need to juggle between what I focus on and where I am present” (55). These accounts suggest that patients with schizophrenia do not simply lose their lifeworld but indeed develop a private world alongside the shared one. Compared to healthy individuals, the boundary between private and shared reality becomes more opaque, producing a dichotomy that is usually less pronounced in mental health, when individual and intersubjective normality overlap more seamlessly.

Finally, a private world need not be interpreted only negatively. It can also carry protective or even emancipatory functions. It may provide a personal niche of resonance (56), serve as protection from aversive emotions (57), or, as Blankenburg suggested, open an eccentric space of individuality (9). In some cases, social withdrawal into a private world is experienced positively, as a form of living in accordance with one’s own eccentric self-evidences (58). A detailed phenomenological case analysis of an individual with schizophrenia suggested that “positive withdrawal” creates a dialectical tension: it involves opening to the shared world while simultaneously maintaining distance from others. Positive withdrawal thus requires a delicate balance of being both inside and outside of society. This may manifest, for example, in selective participation in public spaces that allow presence without demanding intimate social commitment (59).

Thus, the concept of a schizophrenic private world (*Eigenwelt*) captures the ambivalent position of individuals between detachment and participation. On the one hand, a private world (*Eigenwelt*) can appear as an eccentric or even solipsistic retreat. Pushing solipsism too far would, however, result in a collapse of world-constitution. On the other hand, each private world remains inevitably intertwined with shared horizons, drawing on cultural sediments and residual norms. Far from being purely pathological, a private world (*Eigenwelt*) may also function as a protective niche or an eccentric space of freedom.

## 5 A strange world in schizophrenia

### 5.1 Strange in external perception

The flip side of a private world (*Eigenwelt*) would be its external perception as a strange world (*Fremdwelt*), especially when its taken-for-granted assumptions become increasingly idiosyncratic and break with our shared averageness. As integration into the shared normality decreases, it becomes an “other world” or “strange world” (*Fremdwelt*) (38).

Husserl characterizes strangeness as the “verifiable accessibility of what is originally inaccessible” (60). It emerges through the juxtaposition of a persistent core of familiarity with an overall “completely different style [ … ] which extends, iterated analogically, beyond one’s own world” (25). Ultimately, this style may encompass parts that elude every understanding and remain incomprehensible despite all communicative efforts (54).

### 5.2 Mutual alienation and its consequences

A process of mutual, intersubjective alienation between normal and schizophrenic lifeworlds takes place. On the one hand, schizophrenic experiences have been a constitutive part of human society for millennia. However, they are routinely repressed and excluded from our lifeworld so as not to endanger our established taken-for-granted assumptions. This estrangement evokes feelings of unease, threat, and ultimately uncanniness. In line with its psychoanalytic conception, this uncanniness arises from the inaccessibility of experiences that deviate from taken-for-granted assumptions but are nevertheless disturbingly familiar. More so, they are even necessary for constituting normality as a negative imprint.

On the other hand, individuals with schizophrenia themselves become alienated from their premorbid normality. What once felt self-evident begins to appear incomprehensible, until “the social world remains alien” (6). Especially in early phases, the shared lifeworld is experienced as threatening and strange (61). Klaus Conrad described this phenomenon as *apophany*: the intersubjective lifeworld is met with confusion, anxiety, and agitation, eventually giving rise - during acute phases - to delusional interpretations as attempts to make sense of a world that has lost its familiar meanings (6, 61). Even in symptom-poor intervals, many report a persisting sense of isolation and ontological otherness, often as belonging to a “lesser” category of being (62).

These phenomena can also be linked to deficits in social cognition and particularly theory of mind, i.e., the ability to infer others’ mental states (63). However, social perception is likewise impaired: patients with schizophrenia show heightened suggestibility to anger-related cues, which increases feelings of threat and may lead to withdrawal or aggression (63). In casual interactions, they often display reduced facial mimicry of positive emotions, gaze abnormalities, and overall more negative affective expressions (64, 65). On the neurobiological level, this is likely related to disturbed neuronal synchronization with interaction partners in schizophrenia, although such alterations have thus far only been demonstrated in individuals at clinical high risk for psychosis (66). Furthermore, computational neuroscience has discussed predictive coding models as neurobiological correlate of such alienation (67). It is assumed that brain systems continuously generate predictions about incoming sensory and social information and update them in case of prediction errors. In case of schizophrenia, an imbalance between prior expectations and sensory evidence was discussed to increase salience to prediction errors (68). This increased salience is supposed to destabilize the usually smooth anticipation of the world and render it unfamiliar and unpredictable, giving rise to delusions, hallucinations and reduced intersubjective attunement.

The resulting mutual estrangement has concrete social consequences. Central life domains such as romantic partnership or professional life often no longer belong to the taken-for-granted repertoire of many schizophrenic lifeworlds. Disorder-related impairments reduce the ability to “interact appropriately and effectively in the social world” (69). As a result, the experiential horizon narrows, leading to social isolation and decreased satisfaction with the remaining social relationships (70).

In short, the concept of a strange world (*Fremdwelt*) highlights the dialectical estrangement between schizophrenic and shared lifeworlds. From the outside, schizophrenic experiences can appear uncanny and threatening; from the inside, the shared world itself turns strange and hostile. This reciprocal alienation deepens isolation.

## 6 Schizophrenia as part of our lifeworld

### 6.1 Inescapable intersubjectivity

From both the intersubjective dimension of the private world (*Eigenwelt*) and the phenomenon of mutual alienation, it follows that schizophrenic lifeworlds must always be understood as part of our shared world. They cannot be conceived as purely private or self-contained. Rather, the “schizophrenic lifeworld” appears as a shifting constellation between radically anomalous experience and community.

Husserl repeatedly emphasized the inclusive potential of the intersubjective lifeworld. As discussed in Chapter 2, Husserl describes the tension between normality and anomaly as constitutive of every lifeworld. The very existence of anomalous assumptions, however incomprehensible, is a necessary condition for the demarcation of normality (25).

Although the “gap of incomprehensibility” (25) may appear larger in relation to schizophrenia, possible transitions to alien experience nonetheless remain open. Husserl illustrated this in his thought experiment on the relation between the lifeworlds of the normal-sighted and the color-blind, as: (1) dialogical exchange to reconstruct shared cores of subjective experiences; (2) mutual reflection on differences and their psychophysical causes (in the present case e.g. about neurobiological underpinnings of schizophrenia and their effects on perceptual disturbances); (3) mutual attempts to empathically comprehend each other’s world, grounded in our embodiment as human beings, which enables us to re-experience one another’s experience; and (4) reductions of anomalous experiences to our framework of shared normality, enabling at least partial comparison and anticipation of alterations (25, 34).

### 6.2 A fine scale of experiences

Recent developments in user movements (e.g. *Mad Pride*, activist collectives like *Recovery in the Bin*, or the *German Federal Association of (Ex-)Users and Survivors of Psychiatry* (*Bundesverband Psychiatrie-Erfahrener, BPE*)) suggest an even more complex picture of schizophrenic lifeworlds.

Not only do patients within the schizophrenia spectrum inhabit private worlds that are dialectically intertwined with our shared lifeworld, but there is an incredibly fine scale of experiences: some very acute experiences are largely outside the shared lifeworld, some disturbed experiences are at least in partially shared dimensions, and some are even completely shared experiences, sometimes and especially within such peer groups despite the persistence of symptoms.

This implies that individuals with schizophrenia possess very different capacities and resources for entering intersubjective relations with others. Against the background of shared experiences of disorder, many may find it easier to turn to self-help and survivor organizations, where they can also discover a personal niche and experience community even in their individual eccentricity (cf. Chapter 4). Accordingly, empirical studies point towards a modest positive effect of such peer support on personal recovery in mental health (71, 72).

Phenomenology can also benefit from these examples of individuals who, while breaking with large parts of shared experiential normality, nevertheless succeed in creating effective communities, or even “a new culture of madness” (73). This may provide fertile ground for case studies in generative phenomenology to shed light on which pre-reflective processes are essential for establishing shared normality and which can be varied or suspended without preventing the possibility of a shared lifeworld.

### 6.3 The possibility of common ground

Against this backdrop, schizophrenic lifeworlds are not radically strange or impenetrable. Rather, they remain dynamically interwoven with the shared lifeworld, even where communicability is limited. Mutual understanding may require dialogical reconstruction, empathic imagination, or translation into shared frameworks, but it is not foreclosed and a shared core of lifeworld could be constituted (34).

Husserl himself emphasized this inclusive stance: “Finally, also the insane. They are all experienced as ‘psychic’ beings, as ego-subjects, as such indeed also living in the world – the one world – in which we humans live [ … ] as themselves living in the world according to their capacities - in their mode of existence. Every human being—like myself. Each one an ‘Other,’ each one is experienced with understanding as a variation of myself” (34).

All in all, based on the analysis presented in this paper, four interpretations or models of the schizophrenic lifeworld emerge: (1) as a loss of lifeworld, (2) as a private world (Eigenwelt), (3) as a strange world (Fremdwelt), and (4) as part of our shared lifeworld despite marginalization. These four models should not be seen as mutually exclusive but rather as pluralistic and complementary approaches to a better understanding of the schizophrenic lifeworld. For clarity, they are summarized in **Table 1** along with their respective core ideas, sources, and implications. But rather than a mere loss of world or an isolated private world, the “schizophrenic lifeworld” appears as a shifting constellation along a fine scale between isolation and participation, between radical anomaly and shared experience. User movements illustrate how even anomalous experiences can sediment into cultural practices and communities, challenging a purely deficit-oriented view. From the standpoint of Husserl’s generative phenomenology, this demonstrates that the shared lifeworld is capacious enough to include such variations, and that a common ground for dialogue, though fragile, remains possible.

**Table 1:** Models of the schizophrenic lifeworld and their implications.

Model	Core idea	Main sources	Clinical examples	Therapeutic implication
Loss of lifeworld	Radical loss of self-evidence and anchoring in the shared lifeworld	Blankenburg (9) Husserl (19, 25)	Affective flattening; disorganization; temporal disorientation	Search for common ground; attentive presence and constancy
Private world (*Eigenwelt*)	Autism; idiosyncratic self-evidences and eccentric normalities; solipsism in a practical sense, but inevitably intertwined with our shared lifeworld; emancipatory potential (positive withdrawal, personal niche)	Bleuler (49) Fuchs (6) Minkowski (45) Parnas, Sass (10)	Idiosyncratic delusions; “morbid rationalism”	Decoding idiosyncratic meanings; validate protective functions; support positive niches
Strange world (*Fremdwelt*)	Mutual alienation: perceived externally as strange and uncanny, at the same time estrangement and withdrawal of affected individuals	Conrad (61) Husserl (25, 60) Waldenfels (54)	Alienation, uncanniness; deficits in social cognition; disorganization;social isolation	Empathic engagement to reduce alienation; awareness of stigma as “second illness”
Part of our shared lifeworld (but marginalized)	Intertwinement of individual and intersubjective lifeworlds; inclusive potential (dialogue, empathy), but exclusive reality (alienation & stigma)	Husserl (25, 34)	“Double bookkeeping”; user movements	Foster dialogue and community; support inclusive forms of normality

## 7 Discussion

### 7.1 Extending phenomenological psychiatry to intersubjectivity

Schizophrenia must be analyzed not only at the level of subjective disturbance but also within the broader framework of intersubjectivity. Even if, in severe cases, a radical loss of lifeworld, analogous to Blankenburg’s “loss of natural self-evidence”, may appear conceivable, such a notion conflicts with the universality of the lifeworld and risks undermining therapeutic engagement. Instead, people with schizophrenia, like all of us, constitute their own individual lifeworlds, or *Eigenwelten*, which deviate to varying degrees from intersubjective normality. These deviations are not one-sided individual deficits but arise from the interplay between subjective experience, individual lifeworlds, and the shared lifeworld. Husserl’s reflections thus highlight the inclusive potential of the lifeworld.

Hence, Schizophrenia is best understood not as a pure loss of lifeworld, but as a disruption within intersubjective world-constitution that nonetheless presupposes common ground.

### 7.2 Alienation and stigma

In contrast, the reality is often exclusive. Schizophrenic lifeworlds are part of our shared lifeworld, yet they are not merely perceived as strange but become typified in specific ways, e.g. as “human” or the lifeworlds of a “madperson”. What is required for the constitution of a shared lifeworld - reflective and empathetic understanding within a nexus of experience - is frequently absent. Despite the presence of community-based psychiatric services, genuine exchange between psychologically healthy individuals and those with schizophrenia remains rare. Even clinicians, shaped by the dominant “checklist psychopathology” in everyday practice (74), often fail to engage openly with the full subjective experience of those affected.

The causes of this divide are twofold: On the one hand, many people with schizophrenia withdraw from our shared lifeworld, either to live unencumbered in their own taken-for-granted self-relations, or due to deficits in collective intentionality that foster a sense of ontological otherness (62). On the other hand, their participation in our lifeworld is actively curtailed: partly because others lack the imaginative capacity to comprehend anomalous experiences, partly because confrontation with such a disturbing loss of self-evidence threatens the coherence of their own lifeworld. As a result, people with schizophrenia no longer fully participate in the constitution of intersubjective normality. Husserl himself suggested that the intersubjective lifeworld belongs only to those mentally “fully normal”, marked by “rationality and maturity”, while the mentally ill can only participate partially (25).

In this situation, people with schizophrenia are left at the mercy of our shared lifeworld, subjected to typifications of “madness” that reflect stigma more than empathetic understanding. This produces a passivation of those affected. Importantly, such passivation does not arise solely from egological disturbances of their minimal self, but equally from an ecological disruption in the relation between their individual and shared normality. Private worlds in schizophrenia thus arise both because parts of the shared lifeworld are inaccessible due to disorder-specific limitations and because access is actively withheld, whether consciously or unconsciously.

The resulting passive co-functioning within our shared lifeworld can be seen as a resource compared to a total loss of lifeworld. Yet it also entails a particular vulnerability to exclusion and stigmatization. The drastic consequences of stigma for quality of life are well documented (75), frequently described as “second illness” that causes suffering equal to, or greater than, the primary condition itself (76).

### 7.3 Research perspectives

An intersubjective focus can open new research directions. A systemic approach might center on the family structures of those affected (16). Being also conceived as the “psychological agent of society” (77), families represent key interfaces between individual and shared normality. This was also identified by Blankenburg, who in is late works tended the sociocultural and specifically familial context as a risk factor for the development of schizophrenia (16).

Future research should investigate alienation as a mutual process, examining whether suffering arises more from illness-related impairments or from conflicts between individual and intersubjective lifeworlds. Studying transitional phases of psychosis may also allow a reversal of the usual perspective: not only analyzing anomaly from the standpoint of normality, but also interrogating normality from the standpoint of anomaly (28).

The plural and heterogeneous character of schizophrenic lifeworlds, as evidenced in user movements, opens up further avenues for research. These initiatives show that even highly idiosyncratic experiences can become the foundation for collective identities, practices, and traditions. They thus represent exemplary cases of how anomalous experiences may sediment into shared lifeworld formations. Ethnographic and qualitative studies of such movements could provide insights into how individuals with schizophrenia actively reconstruct meaning, negotiate their relationship to the shared lifeworld, and generate new normative frameworks. From the standpoint of generative phenomenology, these empirical contexts are particularly valuable: they make visible how intersubjectivity is reestablished, how new forms of self-evidence take shape, and how “eccentric” experiences can be integrated into broader cultural constellations.

Thus, a critical stance toward intersubjective normality could emerge, that not only improves our understanding of anomalous lifeworlds but also unlocks significant potential for social critique.

### 7.4 Societal implications

At a societal level, the embeddedness of mental illness in the lifeworld implies that anomaly cannot simply be excluded. Broadening our “idea of normality” could foster both self-reflection and inclusion. Importantly, inclusion must not mean assimilation: people with schizophrenia should not be forced into normative molds. Instead, our shared lifeworld must open itself to variation without imposing normalizing pressure. In this sense, Merleau-Ponty’s (1960) notion of “lateral universality” (68) provides a guiding ideal: a reciprocity among diverse lifeworlds, in which anomalous experiences are recognized as variations within the one world we share.

Thus, social inclusion requires a rethinking of normality itself, not as uniformity, but as openness to plural lifeworlds.

### 7.5 Clinical implications

Conceiving of schizophrenia as a wholly private or inaccessible world (*Eigenwelt*) is counterproductive, as it discourages dialogue (38). Therapeutic practice benefits from an explicitly phenomenological stance that suspends normative assumptions and begins by exploring the patient’s world “from within” (78). This requires:

1. Exploration of residual self-evidences: Which taken-for-granted assumptions remain intact? Which new, idiosyncratic ones have emerged, and how might they serve adaptive functions?
2. Attentive presence and constancy: In situations where no bridge to the patient’s lifeworld can be found, the therapist’s regular presence can counteract the risk of radical solitude, or what Gaetano Benedetti called the “desert of the soul” (79). Such presence is especially vital because estrangement from the intersubjective lifeworld often generates profound existential insecurity and an intensified need for hope, reassurance, meaning, and significance - needs that psychotherapy should address explicitly.
3. Establishing tentative bridges of communality: Small, shared routines, embodied forms of resonance, or partial agreements can be used to reopen pathways of communication.
4. Reestablishing routines and relearning trust in the world: Therapeutic work should aim at reestablishing basic routines and gradually rebuilding a sense of trust in the world. Elements from the Soteria approach (e.g. a calm, supportive milieu, continuity of relationships, minimal use of coercion, and embedding of shared daily routines such as cooking), can be especially valuable (80). Empirical studies suggest that the Soteria paradigm may reduce coercive measures and medication doses while maintaining mental health outcomes (81, 82).
5. Balancing normalization and validation: The therapeutic aim is not to force a return to a mythical state of “normal immersion,” but to enable patients to develop sustainable ways of responding to the world, including the integration of eccentric or anomalous experiences into a livable form of existence. Often, patients gradually and often non-linearly relearn how to respond appropriately to environmental demands (83).

### 7.6 Limitations and alternative perspectives

While phenomenology and its therapeutic implications provide important insights, there are also limitations.

Ultimately, biomedical interventions cannot be replaced. Antipsychotic medication is in most cases indispensable for treating acute psychotic symptoms and preventing relapse. While phenomenology contributes conceptual clarity and sensitivity to subjective experience, and while it would be desirable for the therapeutic recommendations outlined above to be more fully realized in clinical practice, phenomenological approaches do not replace standardized treatment guidelines. Rather, they should be understood as a desideratum for more person-centered care.

Moreover, phenomenological psychiatry can be criticized. It could be argued that concepts such as lifeworld are too abstract and difficult to operationalize, both in clinical practice and empirical research. What phenomenology teaches us above all is the rigorous focus on an unprejudiced examination of the subjective experience of those affected, and the sincere effort to build bridges between our shared normality and the full scope of their lived experience. This requires a continuous phenomenological vigilance: a constant effort to bracket our own assumptions while remaining open to the phenomenological value of unusual, and at first seemingly inaccessible, experiences. Translating this attitude into standardized clinical practice is, however, far more challenging. Empirical phenomenological instruments such as the EASE or the EAWE (84, 85) are feasible to assess the basic and world-experience in research contexts, but they remain too complex for routine clinical use and often lack direct therapeutic consequences.

The integration of phenomenological and biological approaches has so far remained sketchy and has been achieved mainly at the level of theoretical models (e.g (2, 3). While empirical realization would be highly desirable, it remains uncertain whether, and how, this could be accomplished without unduly simplifying phenomenological concepts.

## 8 Conclusion

A phenomenological reconsideration of the schizophrenic lifeworld reveals that it cannot be reduced to a simple loss of world or an incomprehensible private sphere. Rather, it unfolds in shifting constellations between isolation and participation. Such a perspective highlights both the vulnerability of people with schizophrenia to exclusion and stigma, and their continuing capacity to contribute to intersubjective meaning and shared normality.

For psychiatry, this perspective carries important implications. Therapeutically, it calls for an attitude of openness and phenomenological vigilance, i.e. entering into dialogue with patients’ lifeworlds without prematurely pathologizing or dismissing their experiential deviations. Scientifically, it suggests the need for further empirical research, including ethnographic and qualitative studies of user movements, as well as attempts to connect phenomenological insights with biological models without reducing their conceptual richness. Societally, it challenges us to expand our idea of normality, fostering more inclusive frameworks that accommodate diverse modes of being-in-the-world.

Ultimately, acknowledging the lifeworld as a shared yet fragile space opens new avenues for an inclusive psychiatry - one that does not merely treat symptoms, but seeks to understand and accompany the human being in their unique way of being-in-the-world.
